# Psychometric Properties of a Questionnaire to Assess Spanish Primary School Teachers’ Perceptions about Their Preparation for Inclusive Education

**DOI:** 10.3390/healthcare10020228

**Published:** 2022-01-25

**Authors:** Jorge Rojo-Ramos, Santiago Gomez-Paniagua, Sabina Barrios-Fernandez, Andres Garcia-Gomez, José Carmelo Adsuar, Jesús Sáez-Padilla, Laura Muñoz-Bermejo

**Affiliations:** 1 Social Impact and Innovation in Health (InHEALTH) Research Group, Faculty of Sport Sciences, University of Extremadura, 10003 Cáceres, Spain; jorgerr@unex.es (J.R.-R.); lauramunoz@unex.es (L.M.-B.); 2 BioẼrgon Research Group, University of Extremadura, 10003 Cáceres, Spain; 3 Occupational Stress, Psychopathologies and Emotional Well-Being (GRESPE) Research Group, University of Extremadura, 06006 Badajoz, Spain; 4 Promoting a Healthy Society Research Group (PHeSO), Faculty of Sport Sciences, University of Extremadura, 10003 Cáceres, Spain; jadssal@unex.es; 5 Faculty of Education, Psychology and Sport Sciences, University of Huelva, 21071 Huelva, Spain; jesus.saez@dempc.uhu.es

**Keywords:** inclusive education, diversity, teachers, perceptions, preparation, primary schools

## Abstract

Inclusive education is a right and must be offered to all students, including those with disabilities, providing them with individualized educational attention and support. Teachers play a leading role in the inclusive education process, their preparation and attitudes being essential for this process. This study aimed to present the factor structure and reliability of a questionnaire for the assessment of teachers’ perceptions about their preparation to support inclusive education. The sample consisted of 605 Spanish primary school teachers who responded to the Evaluation of Teachers’ Inclusion Readiness (CEFI-R) Questionnaire. Exploratory and confirmatory factor analysis and reliability evaluation were performed. The results showed a factor structure with four dimensions (Conception of Diversity, Methodology, Supports and Community Participation), composed of 17 items, with good and excellent goodness-of-fit values and high reliability (Cronbach’s Alpha = 0.75–0.94). Thus, the CEFI-R can be considered a quick and easy-to-apply tool to analyze primary school teachers’ perceptions about their preparation to address their students’ diversity of needs, allowing stakeholders to take actions to promote inclusive education.

## 1. Introduction

Inclusive education is defined as the set of measures that aim to remove or minimize difficulties and barriers that limit the presence, learning and participation of all students [1]. Inclusive schools must create an optimal system that meets the needs of every student and be ready to cope with the different demands that may arise [2]. Inclusive education is a philosophy that requires the educative system to implement and strengthen its principles in different educational communities [2,3,4], in line with rights-based and quality of life approaches in which people with disabilities are recognized as full citizens and therefore subject to accommodation and support [5,6,7]. Moreover, educational policies and practices must support and welcome diversity among all students, not only in terms of functional diversity but also related to ethnicity, social status, sexual diversity and any other form, considering the difference as richness, not a problem [8].

In the field of education, documents such as the Salamanca Declaration [9] and the Incheon Declaration [10] set out a clear stance towards inclusion, proposing frameworks for action towards its achievement. In addition, quality education is the fourth of the 17 Sustainable Development Goals, its aim being to ensure inclusive and quality education for all and promote lifelong learning [11]. In Spain, the current legal reference document is the 8/2013 Organic Law for the Improvement of Educational Quality (LOMLOE), which regulates the structure of the educational system at a statal level [12], dividing the educational system into the following stages: (1) early childhood education from birth to 6 years; (2) primary education, compulsory between 6 and 12 years; (3) secondary education, mandatory until the age of 16, which also includes baccalaureate and professional formation; and (4) university studies. The LOMLOE embraces the Convention on the Rights of Persons with Disabilities and the Convention on the Rights of the Child so no child should be segregated or discriminated against based on his/her disability. Some authors have reflected how the educational systems should be in this century, given the important social changes that are taking place. However, the path towards an inclusive and transformative education will involve overcoming a series of barriers to offer adequate attention to diversity [1,4,13,14,15]. Some of the dimensions that must be considered to achieve inclusive and transformative education include [16]: (1) creating inclusive cultures (building community and establishing inclusive values); (2) establishing inclusive policies (creating a school for all and organizing support for diversity); and (3) developing inclusive practices (building a curriculum for all and orchestrating learning).

Teachers play an important role in achieving educative inclusion; an inclusive teacher should encourage the progression of each student, establishing collaborative and cooperative methods, diversifying his/her teaching methods, encouraging personal autonomy and adapting both the curriculum and the evaluation process and tools [17]. Two of the main factors for the success of inclusion lie in teachers’ preparation [18] and their contact with students with educational needs during this preparation [19]. These factors influence the teachers’ attitudes, which are considered a predictor of diversity acceptance [20]. In addition, self-efficacy, defined as the belief in one’s own abilities to carry out a set of tasks and face the challenges that may arise, is considered to be one of the most important attitude moderators [21,22]. Hence, there is a need to rebuild schools and promote inclusion through consensual decisions and critical reflection [23]. Among the barriers expressed by teachers, one can note their belief that these students are unable to follow normal school classes due to a lack of appropriate materials and assistive technology [24,25] or the lack of initial and ongoing preparation to face the diversity of challenges with these students [26]. 

Subsequently, educational attention to diversity must include actions to prevent inequalities, since they are related to a greater risk of educational failure or school dropout [14]. In this sense, it is essential to generate tools to evaluate aspects linked to educative inclusion [27]. Although there are some instruments that assess inclusive education [12,13], these tools are focused either on the characteristics of students with special needs or on educational practices [28,29,30,31]. The Evaluation of Teachers’ Preparation for Inclusion (CEFI-R) [32] is a widely used questionnaire that can generate an overview of teachers’ perceptions about their preparation to address educative inclusion. However, its factor structure and reliability in the specific context of Spanish primary schoolteachers is unknown. Therefore, this study presents the CEFI-R questionnaire factor structure and reliability to offer an assessment tool to evaluate primary teachers’ perceptions about their preparation to address tasks related to inclusive education and attention to students with diversity. Moreover, exploring the psychometric properties of this instrument will allow us to determine whether it is a valid and reliable tool for stakeholders to undertake actions oriented towards inclusion and diversity. Thus, we asked the following research question: Are the CEFI-R questionnaire’s psychometric properties good enough to assess the Spanish primary school teachers’ perceptions of their preparation to promote inclusive education?

## 2. Materials and Methods

### 2.1. Participants

The sample consisted of 605 primary school teachers from public educative centers in the region of Extremadura (Spain). Their characteristics are shown in Table 1. Their average years of experience amounted to 15.05 years, with a standard deviation of 10.62. Participants were selected using a non-probability convenience sampling method [33]. 

### 2.2. Instruments

A sociodemographic 5-item questionnaire to characterize the sample was designed, with questions about sex, age, the province in which the school is located, the teacher’s type of contract and their years of experience.

The Evaluation of Teachers’ Inclusion Readiness (CEFI-R) Questionnaire was used, which consists of a total of 19 items grouped into four dimensions [32]: (1) Conception of Diversity (5 items), which measures beliefs regarding the concept of diversity, place and form of schooling of students and educational policy on diversity; (2) Methodology (5 items), for aspects related to the design and development of an inclusive curriculum; (3) Supports (4 items), about the teacher’s conception and role in this concept; and (4) Community Participation (5 items), which measures the collaboration of all educational actors. Each item in this instrument is composed of a Likert scale where the values range from 1 to 4, with 1 indicating “Strongly disagree”, 2 “Strongly disagree”, 3 “Strongly agree” and 4 “Strongly agree”. Before the data analysis, indirect items were transposed, so that they coincided with each of the dimensions above. In their original publication, the authors reported a reliability value of 0.79, being >0.70 for each of the four factors [34].

### 2.3. Procedure

To access the sample, an email was sent to the teachers at the public primary schools in Extremadura. To access the schools’ email addresses, the Ministry of Education and Employment of the Regional Government of Extremadura (Spain) was used. The email provided information about the aim of the study, an informed consent form and the link to both the sociodemographic questionnaire and the CEFI-R. They were digitally administered using the Google Forms tool (Google, Mountain View, CA, USA), since e-questionnaires allow greater cost savings, obtaining a higher return and delivery rate [35]. The participants’ responses were stored directly in a spreadsheet for subsequent statistical analysis. Data collection was carried out during September and December 2020.

### 2.4. Data Analysis

The free statistical package FACTOR v.10.10.02 (Rovira I Virgili University: Tarragona, Spain) [36] was used to carry out the exploratory analyses, considering the ordinal nature of the data obtained using a 4-choice Likert scale. The entire sample was split into two equivalent subsamples with the Solomon method [37], using one for the exploratory factor analysis (EFA) and the other for the confirmatory factor analysis (CFA). The robust unweighted least squares (RULS) method with Promin rotation [38] was used for the factor extraction, assuming a correlation between them [39]. Considering the nature of the data, a polychoric correlation matrix [40] was used and the appropriate number of dimensions was established through the optimal implementation of parallel analysis [41]. Once the number of dimensions was identified, the Normalized Direct Oblimin was selected as the rotation method for defining factor simplicity and structure. The Kaiser–Meyer–Olkin (KMO) and Bartlett tests of sphericity were chosen as sampling adequacy indices [42]. 

Subsequently, the software package AMOS v.26.0.0 (IBM Corporation, Wexford, PA, USA) was used to perform the CFA. Elements with loads less than 0.60, with cross loads greater than 0.40 and elements with communalities under 0.30 were deleted [43]. To assess the model’s goodness-of-fit, the following indices were selected: (1) the chi-squared probability setting as appropriate non-significant values (*p* > 0.05) [44]; (2) the comparative fit index (CFI) and (3) the non-normed fit index (NNFI) [45]; (4) the root mean square error of approximation (RMSEA) [46]; (5) the root mean square of residuals (RMSR) [47]; and (6) the chi-square per degree of freedom ratio (CMIN/DF) [48]. In addition, Cronbach’s Alpha coefficient and McDonald’s Omega were selected as the reliability indices [49,50] evaluating the final structure of the questionnaire.

## 3. Results

The RULS method with Promin rotation reported four factors relating to the explained variance based on eigenvalues [51] based on the first half of the sample. The EFA (polychoric correlation matrix can be found in Appendix A) was carried out due to the good results offered by the sampling adequacy indices (Bartlett test = 6875.1; df = 153; *p* = 0.000; and KMO test = 0.83358). Once the number of dimensions was defined, the Normalized Direct Oblimin rotation method was elected considering the necessity of non-parametric techniques due to the level of kurtosis (kurtosis = 46.086; *p* = 0.000). Table 2 shows the rotated loading matrix for 19 items and four factors.

After the EFA, item 11 was excluded as its loading was distributed between two dimensions, Methodology (0.322) and Supports (0.395), introducing high error rates in downstream analyses. Moreover, item 18 was excluded due to negative eigenvalues showing a linear dependence with other items. Therefore, a factor structure of 17 items grouped into four dimensions was extracted.

Table 3 presents the structure and factor loadings of each item (Spanish version can be found in Appendix B). The factor solution was composed of four correlated factors: (1) Conception of Diversity; (2) Methodology; (3) Supports and (4) Community Participation.

Table 4 shows the correlation between CEFI-R questionnaire factors: (1) Conception of Diversity; (2) Methodology; (3) Supports; and (4) Community Participation.

**Figure 1:**
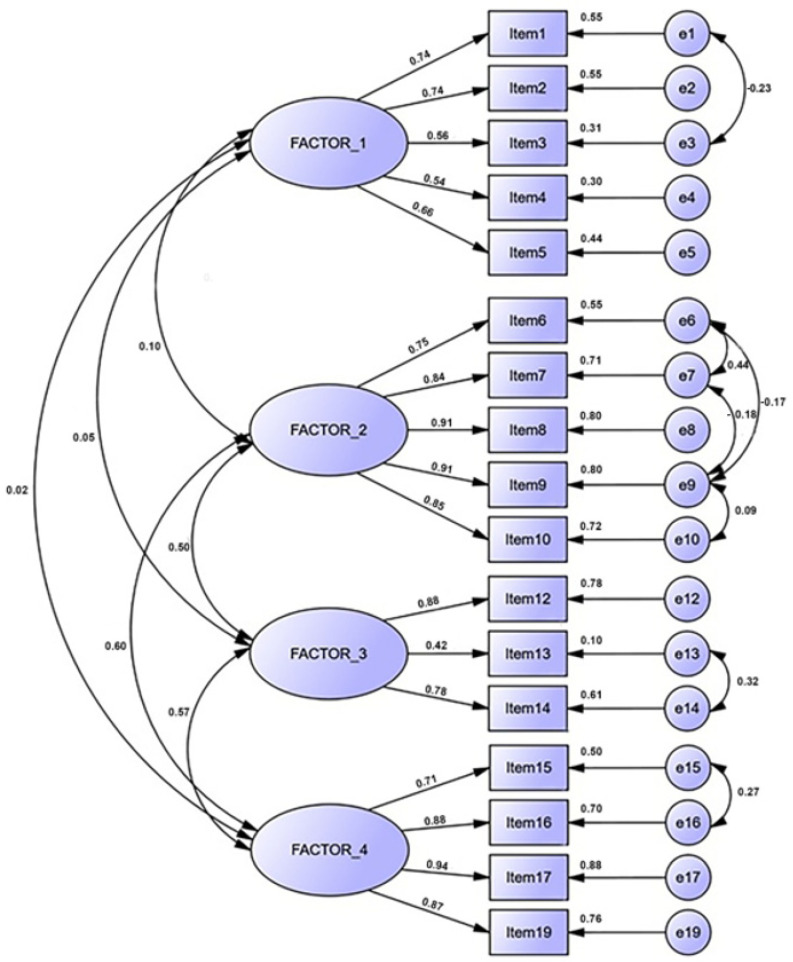
CEFI-R questionnaire factor model.

Once the structure of the questionnaire was defined, the CFA was carried out to establish a definitive model (Figure 1) with the other half of the sample.

Table 5 reflects the CEFI-R goodness-of-fit indices after the CFA [52]. All of them reveal a good fit between the data and the model [53]. The CMIN/DF index shows good values considering that it must be below 2 for a correct model fit, and the chi-squared probability is excellent due to the non-significant values. NNFI and CFI over 0.9 indicate a near-perfect fit to the model. RMSEA is within the established limits (0.010–0.050) and RMSR under 0.08 could be viewed as exceptional.

Table 6 shows reliability indices for the CEFI-R questionnaire dimensions, using the Cronbach´s Alpha, McDonald’s Omega and the explained variance of every factor.

## 4. Discussion

The main contribution of the present study is the investigation of the psychometric properties of the questionnaire to assess teachers’ perceptions about their preparation for inclusive education, providing validity and reliability indicators of the CEFI-R questionnaire in a sample of Spanish primary school teachers. The results showed a factor structure with optimal goodness-of-fit indicators consisting of four interrelated dimensions with 17 items. Furthermore, the Cronbach’s Alpha values showed a high degree of reliability. The four factors that made up the CEFI-R were: (1) Conception of Diversity, as a measure of beliefs about the concept of diversity, place and form of schooling of students and educational policy on diversity [54]; (2) Methodology, including aspects related to the design and development of an inclusive curriculum [55]; (3) Supports, which covers the conceptions and the role of the teacher [56], and (4) Community Participation, measuring the collaboration of all educational agents [57]. Originally, this questionnaire, called CEFI [58], was composed of 80 items grouped into 10 factors. It presented some disadvantages, such as an insufficient sample size and the difficulty to apply a tool with a large number of items. Therefore, a reduced version with 19 items was created [32]. However, the solution of these authors grouped the different items into five factors, even though this meant maintaining some previous shortcomings, such as item 11, deleted in this work due to its generality and its introduction of errors. With regard to reliability, our study is in line with the values presented by the CEFI-R authors [32], showing good and excellent values [49].

Tools such as the CEFI-R will allow educational stakeholders to generate lines of action and programs focused on teachers’ preparation to address their students’ educational needs so that educative inclusion may be achieved [59]. As already mentioned, educational agents’ attitudes are one of the great challenges in the achievement of inclusive education, since they can constitute either a facilitator or a barrier [60,61,62,63,64]. Thus, teachers’ attitudes, composed of three dimensions—cognitive (beliefs), affective (feelings) and behavioral (actions) [60]—are essential to achieve educational quality [2], although it should be noted that sometimes teachers are not sufficiently aware of the diversity of the needs of their students with disabilities [24]. Concerning self-efficacy, it has been reported that teachers with higher levels show greater job satisfaction, while those with lower levels of confidence in their abilities are associated with increased work-related stress and difficulties in coping with their tasks, including dealing with disruptive behaviors [65]. Thus, educational professionals’ preparation influences their attitudes, self-efficacy and their educational practices, so it is necessary to study current and future teachers’ perceptions about their own preparation [64]. According to this, some studies indicate that the curricula of future teachers’ university degrees need to be adapted to comprehensively address the concepts and tools needed to address this transformation [65,66], and ongoing preparation has positive effects on attitudes towards inclusive education [67]. In addition to initial education, ongoing education plays an important role, as teachers must constantly update their preparation to ensure the quality of their educational practices, most importantly regarding students with special educative needs [61,68]. Consequently, knowing in which aspects teachers feel less qualified can help to design formative actions to provide better attention to diversity that allow progress not only towards inclusive quality education but also towards an inclusive society for all citizens [11,69,70].

This research has several limitations. The sample size is limited. All the participants were working in the region of Extremadura, so sociocultural factors could have affected the results. This is preliminary work, as the validation of an instrument is a process that is built up over time. Future work is needed to provide further evidence of the CEFI-R’s psychometric properties. This research does not use direct data collection methods that present more valid results than telephone or online surveys [71]. In contrast, online questionnaires have the advantages from the researcher’s point of view of reducing costs, relocating the interviewer concerning the respondents, enlarging the sample and facilitating data collection and processing. Additionally, these results only apply to primary schoolteachers. As future lines of research, recruiting a larger sample, from different regions in Spain, would be interesting to obtain further evidence on the CEFI-R’s strengths. Using the CEFI-R at different educational stages and with diverse educational stakeholders could be of interest to understand the perceptions of those involved in inclusive education.

## 5. Conclusions

The current research presents validity and reliability indicators of a questionnaire to assess teachers’ perceptions about their preparation to address educative inclusion: the CEFI-R questionnaire. According to our results, a solution composed of 17 items explained by four factors shows consistent goodness-of-fit indicators and good and excellent reliability values. This questionnaire is suitable for educational and research purposes in primary schools and takes no more than three minutes to administer, being a free and easy-to-use tool.

Analyzing teachers’ perceptions of their training needs is an important aspect as it influences their attitudes and self-efficacy, which in turn is reflected in how they handle attention to diversity, and therefore whether or not they implement educational practices that promote the transition to an inclusive and transformative school.

## Figures and Tables

**Table 1:** Sociodemographic characteristics of the sample (*n* = 605).

Sex	Age	Province			
		Caceres		Badajoz	
		Teacher Type of Contract			
		Temporal	Indefinite	Temporal	Indefinite
Men	Under 30	2	0	9	2
	Between 30 and 40	22	7	17	22
	Between 41 and 50	7	16	1	31
	Over 50	0	10	2	22
Women	Under 30	9	1	20	3
	Between 30 and 40	28	20	43	62
	Between 41 and 50	6	33	19	87
	Over 50	1	19	9	75

**Table 2:** Rotated loading matrix with Normalized Direct Oblimin.

Item	Factor 1	Factor 2	Factor 3	Factor 4
1. I would prefer to have students with specific educational support needs in my classroom.	0.754	0.010	0.058	0.023
2. A child with specific educational support needs does not disrupt the classroom routine and disrupt the learning of his/her classmates.	0.767	−0.015	−0.008	0.042
3. We should place students with special educational needs in mainstream schools even if we do not have the appropriate preparation.	0.608	0.030	0.024	−0.119
4. Students with specific educational support needs can follow the day-to-day curriculum.	0.681	−0.001	−0.118	0.064
5. I am not worried that my workload will increase if I have students with specific educational support needs in my class.	0.682	−0.004	0.060	0.029
6. I know how to teach each of my students differently according to their characteristics.	−0.031	0.868	−0.009	−0.043
7. I know how to design teaching units and lessons with the diversity of students in mind.	−0.006	0.972	−0.037	−0.060
8. I know how to adapt the way I assess the individual needs of each of my students.	0.034	0.941	0.010	0.003
9. I know how to handle and adapt teaching materials to respond to the needs of each of my students.	0.030	0.908	−0.002	0.066
10. I can adapt my communication techniques to ensure that all students can be successfully included in the mainstream classroom.	0.031	0.818	0.063	0.066
11. Joint teacher-support teacher planning would make it easier for support to be provided within the classroom.	−0.055	0.383	0.366	0.295
12. I believe that the best way to provide support for students is for the support teacher to be embedded in the classroom, rather than in the support classroom.	−0.050	0.090	0.130	0.749
13. The role of the support teacher is to work with the whole class.	0.049	0.010	−0.058	0.541
14. I consider that the place of the support teacher is in the regular classroom with each of the teachers.	−0.042	−0.050	0.095	0.907
15. The educational project should be reviewed with the participation of the different agents of the educational community.	0.033	−0.020	0.776	0.110
16. there must be a very close relationship between the teaching staff and the rest of the educational agents.	0.051	−0.080	0.994	−0.025
17. The school must encourage the involvement of parents and the community.	0.011	0.027	0.961	−0.017
18. Each member of the school (teachers, parents, students, other professionals) is a fundamental element of the school.	−0.061	0.126	0.897	0.036
19. The school must work together with the resources of the neighbourhood.	−0.021	0.048	0.917	−0.025

Note: These items are a literal translation into English for ease of reading, not a cross-cultural adaptation into English.

**Table 3:** CEFI-R questionnaire rotated factor solution and factor loading.

Items	Factor 1	Factor 2	Factor 3	Factor 4
1. I would prefer to have students with specific educational support needs in my classroom.	0.671			
2. A child with specific educational support needs does not disrupt the classroom routine and disrupt the learning of his/her classmates.	0.693			
3. We should place students with special educational needs in mainstream schools even if we do not have the appropriate preparation.	0.642			
4. Students with specific educational support needs can follow the day-to-day curriculum.	0.782			
5. I am not worried that my workload will increase if I have students with specific educational support needs in my class.	0.668			
6. I know how to teach each of my students differently according to their characteristics.		0.882		
7. I know how to design teaching units and lessons with the diversity of students in mind.		0.976		
8. I know how to adapt the way I assess the individual needs of each of my students.		0.957		
9. I know how to handle and adapt teaching materials to respond to the needs of each of my students.		0.899		
10. I can adapt my communication techniques to ensure that all students can be successfully included in the mainstream classroom.		0.789		
11. Joint teacher-support teacher planning would make it easier for support to be provided within the classroom.	Deleted			
12. I believe that the best way to provide support for students is for the support teacher to be embedded in the classroom, rather than in the support classroom.			0.752	
13. The role of the support teacher is to work with the whole class.			0.458	
14. I consider that the place of the support teacher is in the regular classroom with each of the teachers.			0.864	
15. The educational project should be reviewed with the participation of the different agents of the educational community.				0.568
16. there must be a very close relationship between the teaching staff and the rest of the educational agents.				0.917
17. The school must encourage the involvement of parents and the community.				0.686
18. Each member of the school (teachers, parents, students, other professionals) is a fundamental element of the school.	Deleted			
19. The school must work together with the resources of the neighbourhood.				0.930

Note: These items are a literal translation into English for ease of reading, not a cross-cultural adaptation into English.

**Table 4:** CEFI-R inter-factor correlation matrix.

Factors	Factor 1 Conception of Diversity	Factor 2 Methodology	Factor 3 Supports	Factor 4 Community Participation
Factor 1 Conception of Diversity	1			
Factor 2 Methodology	0.143	1		
Factor 3 Supports	0.041	0.343	1	
Factor 4 Community Participation	−0.056	0.524	0.517	1

**Table 5:** CEFI-R questionnaire goodness-of-fit indices.

Indices	Value
CMIN/DF	1.719
Ρ (*χ*^2^)	0.99
NNFI	0.943
CFI	0.974
RMSEA	0.048
RMSR	0.042

CMIN/DF: minimum discrepancy per degree of freedom; Ρ (χ2): chi-squared probability; CFI: comparative fit index; NNFI: non-normed fit index, RMSEA: root mean square error of approximation; RMSR: root mean square of residuals.

**Table 6:** Internal consistency of the CEFI-R questionnaire.

Indexes	Factor 1 Conception of Diversity	Factor 2 Methodology	Factor 3 Supports	Factor 4 Community Participation
Cronbach’s Alpha	0.94	0.94	0.76	0.75
McDonald’s Omega	0.82	0.95	0.74	0.89
Explained Variance	2.43	4.46	1.98	3.27

## Data Availability

The datasets used during the current study are available from the corresponding author on reasonable request.

## Appendix A

**Table A1:** Polychoric correlation matrix extracted from the EFA.

Items	1	2	3	4	5	6	7	8	9	10	15	16	17	19	12	13	14
1	1.000																
2	0.632	1.000															
3	0.378	0.452	1.000														
4	0.484	0.526	0.470	1.000													
5	0.553	0.476	0.441	0.462	1.000												
6	0.115	0.057	0.006	0.033	0.057	1.000											
7	0.135	0.038	0.086	0.053	0.107	0.855	1.000										
8	0.173	0.142	0.078	0.038	0.129	0.779	0.868	1.000									
9	0.177	0.097	0.071	0.052	0.160	0.762	0.839	0.927	1.000								
10	0.137	0.120	0.085	0.020	0.163	0.727	0.795	0.835	0.855	1.000							
15	0.067	0.048	−0.053	−0.005	0.125	0.327	0.361	0.448	0.485	0.467	1.000						
16	0.136	0.119	−0.022	0.063	0.071	0.170	0.179	0.187	0.203	0.223	0.414	1.000					
17	0.088	0.036	−0.057	0.053	0.077	0.286	0.291	0.341	0.397	0.381	0.792	0.506	1.000				
19	0.094	0.032	0.011	−0.102	0.114	0.432	0.465	0.503	0.485	0.523	0.520	0.240	0.515	1.000			
12	0.091	−0.023	−0.005	−0.077	0.078	0.479	0.515	0.574	0.588	0.591	0.573	0.233	0.517	0.911	1.000		
13	0.044	−0.043	−0.052	−0.098	0.039	0.547	0.580	0.639	0.645	0.656	0.637	0.199	0.579	0.907	0.954	1.000	
14	0.019	−0.012	−0.026	−0.071	0.054	0.482	0.498	0.565	0.587	0.570	0.545	0.195	0.517	0.877	0.910	0.949	1.000

## Appendix B

**Table A2:** Cuestionario para la Evaluación de la Preparación del Profesorado para la Inclusión (CEFI-R). Reprinted from ref. [61].

1. Preferiría no tener en mi aula alumnos con necesidades específicas de apoyo educativo
2. Un niño con necesidades específicas de apoyo educativo interrumpe la rutina del aula y perjudica el aprendizaje de sus compañeros
3. No debemos escolarizar alumnos con necesidades educativas especiales en centros ordinarios hasta que no tengamos la formación adecuada para ello
4. Los alumnos con necesidad específica de apoyo educativo no pueden seguir el día a día del curriculum
5. Me preocupa que mi carga de trabajo se incremente si tengo alumnos con necesidades específicas de apoyo educativo en mi clase
6. Sé cómo enseñar a cada uno de mis alumnos de manera diferente en función de sus características individuales
7. Sé cómo elaborar las unidades didácticas y las clases teniendo presente la diversidad de los estudiantes
8. Sé cómo adaptar mi forma de evaluar a las necesidades individuales de cada uno de mis alumnos
9. Sé cómo manejar y adaptar los materiales didácticos para responder a las necesidades de cada uno de mis alumnos
10. Soy capaz de adaptar mis técnicas de comunicación para asegurarme de que todos los alumnos puedan ser incluidos con éxito en el aula ordinaria
11. La planificación conjunta profesor-profesor de apoyo facilitaría que los apoyos se proporcionaran dentro del aula
12. Creo que la mejor manera de proporcionar apoyo a los alumnos es que el profesor de apoyo se incorpore al aula, en lugar de hacerlo en el aula de apoyo
13. La función del profesor de apoyo es trabajar con todo el alumnado de mi aula
14. Considero que el lugar del profesor de apoyo está dentro del aula ordinaria con cada uno de los profesores
15. El proyecto educativo debería revisarse con la participación de los distintos agentes de la comunidad educativa (profesores, padres, alumnos...)
16. Es fundamental que haya una relación muy estrecha entre el profesorado y el resto de agentes educativos (AMPA, asociación de vecinos, consejo escolar...)
17. La escuela debe fomentar la implicación de los padres y de la comunidad
18. Cada miembro del centro educativo (profesores, padres, alumnos, otros profesionales) es un elemento fundamental del mismo
19. El centro debe trabajar de forma conjunta con los recursos del barrio (biblioteca, servicios sociales, servicios sanitarios...)
